# The Model of Empathic Pain Assessment and Treatment in Persons With Dementia

**DOI:** 10.1093/geroni/igaa057.1701

**Published:** 2020-12-16

**Authors:** Lauren Starr, Kristin Corey Magan

**Affiliations:** 1 University of Pennsylvania School of Nursing, Philadelphia, Pennsylvania, United States; 2 University of Rhode Island College of Nursing, Providence, Rhode Island, United States

## Abstract

There are 5.8 million people with Alzheimer’s dementia in the United States—81% are 75 years or older. Although half of persons with dementia regularly experience pain, their pain is underrecognized and undertreated, partly because clinicians experience challenges assessing pain in persons with dementia who cannot self-report. Evidence suggests clinician empathy is involved in pain assessment and treatment. Conceptual models guiding Alzheimer’s research are lacking in the literature. To create an interdisciplinary, evidence-based model for understanding clinical empathy’s relationship with the assessment and treatment of pain in persons with advanced dementia, we conducted a literature review of relevant manuscripts from 2000-2019 across disciplines and countries, emphasizing dementia studies and research conducted in the last decade. After performing quality appraisal using the Oxford Centre for Evidence-based Medicine’s levels of evidence, we synthesized findings from 38 qualifying studies and developed a new conceptual model driven by observation of behaviors indicating pain in persons with dementia unable to self-report. The model represents the cognitive, affective, ethical, and behavioral components of clinical empathy involved in assessing and treating pain, relevant patient outcomes, and contextual factors influencing empathy and outcomes; and provides a framework for testing clinical empathy interventions to improve adverse outcomes in persons with advanced dementia. Understanding the relationship between clinician empathy and the assessment/treatment of pain in persons with dementia may improve care quality and help reduce pain behaviors in this population. This model may be used to inform pain research in persons with dementia and develop clinical interventions and clinician education programs.

